# Increased prevalence of oral potentially malignant lesions among Croatian War invalids, a cross-sectional study 

**DOI:** 10.4317/jced.60715

**Published:** 2023-09-01

**Authors:** Livia Cigic, Dinko Martinovic, Jure Martinic, Mare Kovic, Ana Druzijanic, Ivan Galic, Antonija Tadin, Bozanela Lukanovic, Mirna Duzel, Tina Poklepovic-Pericic

**Affiliations:** 1 Department of Maxillofacial Surgery, University Hospital of Split, 21000 Split, Croatia; 2 Study of Dental Medicine, School of Medicine, University of Split, 21000 Split, Croatia; 3 Dental Medicine Office Stanko Krnjic, 20000 Dubrovnik, Croatia; 4 Dental Medicine Office Franka Radic Jakir, 21300 Makarska, Croatia

## Abstract

**Background:**

The main objective of this study was to investigate the frequency and type of oral pathological changes, oral subjective symptoms and the knowledge about oral cancer in the population of Croatian military invalids from the Homeland War.

**Material and Methods:**

A total of 102 Croatian military invalids from the Homeland War participated in the study. Data were collected on the presence of subjective symptoms in the oral cavity, and a detailed clinical examination of the oral mucosa was performed.

**Results:**

Almost half of the participants, 46 (45.1%), reported being smokers, and 64 (62.7%) consumed alcohol daily. Subjective symptoms in the oral cavity were reported by 25 (24.5%) of them. Pathological changes were found in 35 (34.3%) participants, of whom 14 (13.7%) had potentially malignant changes. Pathohistological findings confirmed the diagnosis of a potentially malignant lesion in 10 subjects and indicated the presence of moderate dysplasia in two, carcinoma in situ in one, and invasive carcinoma in one.

**Conclusions:**

Participants didn’t show adequate knowledge of risk factors. Forty-one changes in the oral cavity were found in 35 subjects, and as many as 14 were potentially malignant. According to the participants, most dentists and family physicians don’t thoroughly and regularly examine their patients’ oral mucosa.

** Key words:**Oral cancer, oral lichen planus, leukoplakia, erythroplakia, actinic cheilitis, Croatian Homeland War invalids.

## Introduction

Croatian war invalids from the Homeland War (1990-1995) belong to the risk group for developing oral cancer, both because of the war’s psychological consequences and everyday bad habits, such as drinking alcohol and smoking cigarettes. According to the Croatian Encyclopedia, the Homeland War was a defensive war for the independence and integrity of the Croatian state against the aggression of the united Greater Serb forces (1). Considering they were exposed to stressful and traumatic events during the war and are consequently believed to be more inclined to smoking cigarettes and drinking alcohol. That is why, together with their age and gender, they represent a specific risk group for developing potentially malignant changes in the oral cavity (2).

Oral cavity cancer is one of the ten most common cancers in humans. In 90% of cases, it is a squamous cell carcinoma, a malignant tumour of the multilayered mucosal epithelium. In the remaining 10% of cases, it is a malignant tumour of the salivary glands, melanomas, verrucous carcinomas, sarcomas, and metastatic tumours (2). Oral cancer is characterized by high mortality due to local spread, regional and distant metastases, and the usually advanced stage of the disease at treatment initiation (3,4). The highest percentage (80%) of oral cavity cancer is located in the area known as the horseshoe (floor of the oral cavity, lateral margins of the tongue, ventral surface of the tongue and retromolar region, and palatal arches). Oral cavity cancer most commonly occurs in the middle-aged or older male population. However, as more and more women become addicted to alcohol and tobacco, the incidence of oral cavity cancer also increases in the female population (5,6). Smoking and alcohol are among the most important etiologic factors for developing oral cavity cancer (2). Their synergistic effect is also significant. Other factors influencing cancer development include poor socioeconomic status, poor oral hygiene, older age, a diet containing nitrosamines, and vitamin deficiency. Ultraviolet radiation is also a crucial etiological factor in developing lip cancer (2,3). The influence of human papillomavirus, Epstein-Barr virus, and **Candida* albicans* infection on the occurrence of oral cavity cancer is increasingly being studied (5). According to the World Health Organization definition (WHO), potentially malignant changes or precancerous lesions are divided into precancerous lesions and precancerous conditions (7). A precancerous lesion is a morphologically altered tissue in which cancer is more likely to develop than in apparently unchanged mucosa. This group includes leukoplakia, erythroplakia, oral lichen planus, and actinic cheilitis (7).

Precancerous conditions are disorders of the general state of the organism with an increased risk of developing cancer, e.g. Plummer-Vinson syndrome. Oral cavity cancer is initially completely asymptomatic, so in many patients, it is discovered only after the onset of symptoms, when the disease is already advanced. The first symptom is a nonspecific malaise in 85% of patients (3). Symptoms most common in the advanced stage of the disease include odynophagia, dysphagia, otalgia, limited jaw mobility, and bleeding from the oral cavity. The appearance of the lesion may be red, white, or red-white, apartment or raised, ulcerated or non-ulcerated, and the lesion may also be minimally palpable or indurated. Ulceration that does not heal within 14 days and does not respond to therapy requires further treatment. It is characteristic of these malignancies to metastasize early to the cervical lymph nodes, so it is important to be alert to the presence of swelling in the neck. When diagnosing oral cavity cancer, it is necessary to take a detailed history, examine the entire oral cavity and palpate the lymph nodes of the head and neck, the floor of the oral cavity and the tongue (3,4).

Other diagnostic methods that allow the diagnosis of oral cavity cancer include biopsy of the lesion and histopathological findings, cytodiagnosis, and diagnostic imaging. The degree of extension of the tumour is the essential factor in the prognosis of the disease and the choice of treatment modality. Treatment usually includes surgery and postoperative radiation (4,5,8).

Global data on cancer (GLOBOCAN) show that in 2020, 377,713 people were diagnosed with oral cancer worldwide, and 177,757 died from this disease (9). In addition, according to the Croatian Institute of Public Health, 633 people in Croatia were diagnosed with oral cavity and pharynx cancer in 2020 (10).

The main aim of this study was to investigate the frequency and nature of pathological changes in the oral mucosa and the presence of subjective symptoms in the oral cavity in the population of Croatian military invalids from the Homeland War and to test their attitudes and knowledge about oral cancer.

## Material and Methods

-Study design

This cross-sectional study was conducted in the Department of Maxillofacial Surgery, University Hospital of Split, from April 2018 to April 2020.

All included participants were informed in time about the purpose and aim of the study. In addition, they all gave their written informed consent to participate before the start of the study. The study was approved by the Ethics Committee of the University of Split School of Medicine and was conducted in accordance with the current revision of the Declaration of Helsinki.

-Subjects

Participation in this study was voluntary, and all Croatian invalids of the Home-land War were recruited through veterans’ organizations and associations. The inclusion criteria were: 18-70 years old; disability due to the Homeland War. We used a convenient sample. The exclusion criteria were: active psychotic illness or legal loss of decision-making capacity. A total of 114 potential subjects agreed to take part in this study and were screened. However, 12 of them were excluded because they did not meet the study criteria. Thus, 102 disabled Croatian war veterans were included in this study.

-Instruments

After voluntarily agreeing to participate in the study, all participants were enrolled for a detailed physical examination of the oral cavity at the Department of Oral Medicine, University Hospital of Split.

During their examination, they were first given an anonymous questionnaire. The questionnaire was adopted by the Croatian Society of Oral Medicine with their approval and structured according to the latest relevant literature. The questionnaire contains four questions on general and demographic information. There are also 11 questions on daily habits, such as cigarette smoking and alcohol consumption. The last part of the questionnaire concerns knowledge about oral cavity cancer. It consists of five items rated on a five-point Likert scale and five items with multiple-choice answers.

After completing the questionnaire, a detailed medical history was obtained. All respondents were asked how often they visited the dentist and primary care physician and whether they were examined regularly to detect early forms of oral cavity cancer and potentially malignant changes. Data were also collected on the presence and nature of subjective oral cavity disorders.

Next, an experienced oral medicine specialist performed a detailed clinical examination of the oral mucosa to identify and record pathological changes in the oral mucosa, its size and location. Moreover, the subjects in whom the clinical examination revealed a suspicious lesion came to the Department of Maxillofacial Surgery, University Hospital of Split for an additional examination to perform a tissue biopsy for pathohistological analysis. After local anaesthesia, a sample of the pathologically altered oral mucosa was obtained by biopsy, and the pathohistological diagnosis was made by a specialized pathologist of the Department for Forensic Medicine, Pathology and Cytology, University Hospital of Split.

-Statistical data analysis

Statistical analysis was performed with the statistical package MedCalc for Windows (MedCalc Software version 19.4., Ostend, Belgium). First, the normality of the distribution was estimated using the Kolmogorov-Smirnov test. Quantitative variables were presented either as mean ± standard deviation or median (interquartile range), depending on the normality of data distribution. Categorical variables were expressed as whole numbers and percentages.

## Results

-General characteristics of the participants

The study included 102 Croatian war invalids, of whom 88 (86.3%) were men and 14 (13.7%) were women. The mean age was 54.3 years, the minimum was 44 years, and the maximum was 75 years. Regarding work status, 91 (89.2%) of the participants indicated that they were retired, eight (7.8%) were employed, and three stated that they were unemployed (2.9%). Regarding the participants’ education, most have secondary vocational education (77 of them or 75.6%). Fourteen of them (13.7%) have higher education, eight respondents (7.8%) have a college education and three of them (2.9%) have completed only elementary school. The majority of the participants, 82 (80.4%), indicated the city as their residence, while the remaining 20 (19.6%) stated the countryside.

-Habits

Overall, the study sample included 46 smokers (45.1%), while 56 (54.9%) denied smoking cigarettes daily. The average number of cigarettes smoked daily per smoker was 24, while the average number of years of cigarette use was 29. In response to the question, “Are you considering quitting smoking?” 19 out of 46 smokers (41.3%) said they planned to quit smoking in the next six months. Three respondents (6.5%) stated that they are currently quitting smoking, while 24 (52.2%) do not plan to quit smoking.

In addition, 64 respondents (62.7%) consume alcohol daily, while 38 (37.3%) deny alcohol consumption. Almost half of the respondents (43.7%) who drink alcohol answered in the affirmative to the question “Have you ever thought about limiting your alcohol consumption?”, while 34.4% of them feel guilty about their alcohol consumption.

-Subjective symptoms and clinical findings

**Figure 1:**
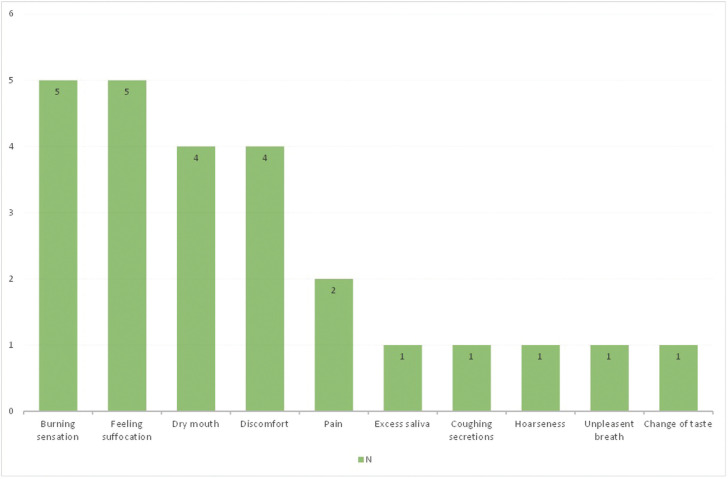
Frequency and type of subjective symptoms.

The majority of participants, 77 (75.5 %), deny the existence of subjective symptoms in the oral cavity, while 25 (24.5 %) state the following: burning five, feeling of suffocation also five, xerostomia four, discomfort four, pain two, and one respondent reported increased salivation, coughing up secretions, hoarseness, foetor ex ore and a change in taste sensitivity (dysgeusia) (Fig. 1).

A detailed clinical examination revealed normal clinical findings of the mucosa in 67 (65.7%) subjects, while a total of 41 changes in the oral cavity were found in 35 (34.3%) subjects. Leukoedema of the cheek was observed in five subjects, which is not considered a pathological change but a normal morphological change of the mucosa. Of the remaining 36 changes, the most common finding was related to decreased salivary secretion - in seven of 35 subjects with changes (20%), the oral mucosa was dry, and in two subjects (5.7%), the tongue was fissured and sticky. Depending on the location, the most frequent changes were found on the tongue mucosa (8 out of 36 changes): black hairy tongue in four, white hairy tongue in three, and geographic tongue in one subject. One papilloma was diagnosed in the cheek area, while the consequences of biting on the mucosa were also noted in four subjects - traumatic fibroma in the interocclusal line on the cheek and patchy epithelial deposits on the cheek (morsicatio) in two subjects. Pathologic changes corresponding to potentially malignant changes were found in 14 subjects (13.7%), of which four subjects (3.9%) had hyperkeratotic changes suspicious for leukoplakia. In addition, lesions consistent with oral lichen planus were found in four subjects, actinic cheilitis in three subjects, and changes suggestive of erythroplakia in also three subjects (Table 1).

**Table 1:**
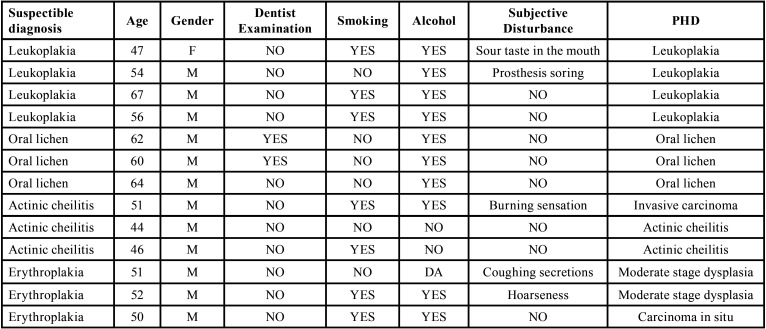
Characteristics of subjects with potentially malignant changes on the mucous membrane of the oral cavity.

Histopathological findings confirmed the clinical suspicion in all cases of leukoplakia and oral lichen planus. In the case of actinic cheilitis, the diagnosis was confirmed in two patients, while in one patient, the histopathological findings showed that it was squamous cell carcinoma.

In the case of erythroplakia, the findings in two subjects showed the presence of dysplasia of moderate extent, and in one subject, the pathohistological findings revealed a carcinoma in situ.

-Participants’ awareness of oral cavity cancer

The majority of participants, 62 of them (60.7%), indicated their environment as a source of information about oral cancer, 31 participants (30.4%) obtained information from the media, one participant (1%) indicated that they had educated themselves, while as many as six (5.9%) had never heard of oral cancer. The remaining two participants stated that they did not know the source of information about oral cavity cancer.

-Preventive examinations of the oral cavity

When asked about a regular examination of the entire oral cavity for early detection of potentially malignant changes in the oral cavity, 33 (32.4%) of the 102 participants answered that their dentist performs this type of examination regularly. However, as many as 69 participants (67.6%) indicated that their selected dentist must complete or perform the examination in detail. As for the selected general practitioners, only seven (6.9%) answered that their general practitioner examines the mucosa of the oral cavity. When asked about the frequency of visits to the selected dentistry doctor, most of the respondents answered that they go for a regular examination once a year (40 of them, i.e. 39.2%), while as many as 14 (13.7%) have not had an examination for more than two years.

-Knowledge and attitudes about oral cancer

The participants’ knowledge of risk factors for the development of oral cavity cancer is shown in Table 2.

**Table 2:**
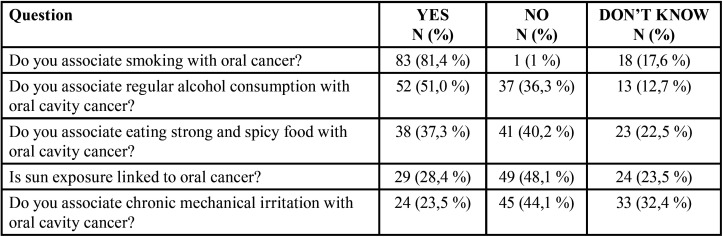
Participants’ knowledge of risk factors for the development of oral cavity cancer.

Most participants, 56.5%, believe that the incidence of oral cancer is the same compared to other cancers. The same percentage of participants believe that the mortality of oral cancer is the same compared to other cancers. In addition, the most significant portion of participants (48.5%) believe they have the same chance of developing oral cancer in the future compared to others of their age and gender. Of the participants who self-identified as smokers, the most significant percentage (61.6%) believe that they have the same chance of developing oral cancer compared to other smokers, and compared to other nonsmokers, 38.3% of them think that their chances are also equal and not greater.

No participant (0%) recognized the correct combination of possible early symptoms of the disease (loss of taste, dry mouth, bleeding gums, burning sensation in the mouth, numbness of the tongue and surrounding regions, difficulty chewing and swallowing, unusual swelling in the mouth, painful changes in the mouth that bleed and do not heal, loose teeth and tooth loss, constant pain in the jaw, white or red changes in the gums) from the answers given.

The majority of participants, 74 (72.5%), believe that the risk of oral cavity cancer increases with age, while 74 (72.5%) also believe that cancer is not an infectious disease. Eighty-one participants (79.4%) believe oral cavity cancer can be prevented, and 82 (80.4%) believe cancer treatment is possible. Most participants (37.6%) chose from the offered options for mortality from oral cavity cancer that it is 6% to 25%.

## Discussion

Croatian war-disabled veterans are a specific risk group due to long-term exposure to stress and traumatic events during the Homeland War, which, in addition to frequent bad daily habits of alcohol consumption and cigarette smoking, has been proven to lead to more frequent development of post-traumatic stress disorder (PTSD) (4,12). Searching the literature, we did not find published results of scientific research on the same topic, and it seems that this is the only research that covered the knowledge and attitudes of this specific group in the Republic of Croatia about oral cavity cancer.

Muhvić-Urek *et al*. showed that Croatian war invalids from the Homeland War diagnosed with post-traumatic stress disorder practice poor oral hygiene and have more frequent diseases and disorders related to the oral cavity (13). The increased risk for developing pathological changes in the oral cavity results from several risk factors in this group - average age, gender, and tendency to daily bad habits such as smoking cigarettes and drinking alcohol. It has been proven that oral cavity cancer appears more often in men with an average age of around 60 years, although recently, the frequency has been increasing in women and people of a younger age (9,14,15).

 A total of 102 participants voluntarily participated in this research, of which 88 (86.3%) were men with an average age of 54.3 years. Toscano de Brito and colleagues also confirmed in their study that in men over 60 years of age, the incidence of oral cavity cancer increased, while the clinical stage of the disease was not related to age (16).

Daily habits of the included subjects have been proven to contribute to cancer development (14,17). We researched a specific group of at-risk participants, among whom 62.7% answered that they consume alcohol daily, while 45.1% of the included subjects smoke cigarettes daily. Among participants who drink alcohol, the majority (65.6%) do not feel guilty about their consumption, while more than half (56.3%) do not think they should reduce their alcohol consumption. On average, they smoke more than a pack of cigarettes a day (24 cigarettes), and the average number of years they smoke is 29. It is worrying that more than half of the participants do not even consider quitting smoking. The data mentioned above confirm the thesis about the propensity of the population of Croatian war invalids to harmful habits, which is why they indeed belong to the group with an increased risk of developing oral cavity cancer (4,8). Through a study in Germany, Mons and colleagues estimated the absolute number and proportion of cancers that can be directly linked to smoking and regular alcohol consumption. The most common form of cancer associated with smoking is lung cancer, where 89% of male subjects and 83% of female subjects confirmed that they smoke daily. Alcohol consumption is associated with oral cavity, oesophagus and pharynx cancer. Since a substantial proportion of cancers can be directly linked to the aforementioned factors, exceptional efforts are needed in the prevention of smoking and alcohol consumption to consequently reduce the frequency of related forms of cancer (18). In a study conducted in 2016 in Arab countries, a strong connection between oral cavity cancer and smoking cigarettes and smokeless tobacco products was proven, while alcohol consumption and exposure to sunlight are possible factors for its occurrence. The most common localizations of pathological changes were the tongue, the oral cavity floor, and the lower lip (19).

Participants’ views on the harmful habits of smoking and alcohol consumption are interesting: 17.6% of participants do not know that smoking is a risk factor for developing oral cavity cancer, while 12.7% do not know about daily alcohol consumption. In addition, 36.3% of participants believe that regular alcohol consumption does not contribute to cancer risk. Furthermore, exposure to the sun is recognized as a risk factor by only 28.4% of participants and chronic mechanical irritation by 23.5%. However, studies have proven that this is a factor in developing pathological changes in the oral mucosa (20). In addition, only 28.8% of smokers believe that they have a higher risk of developing oral cavity cancer than nonsmokers, while the other smokers believe that their chances are equal or lower. These observations are consistent with the results of previous studies. They indicate the need to educate both Croatian war invalids and the general population to raise awareness of the harmful effects of alcohol consumption and cigarette smoking, especially the synergistic effect of these habits on the occurrence of pathological changes in the oral cavity mucosa (21,22).

When asked from whom they had heard about oral cavity cancer, 60.7% of the participants indicated that the environment (friends, colleagues, family) was their primary source of information, while 5.9% had never heard of oral cavity cancer. However, the accuracy of information from the environment is questionable. Park *et al*. conducted a survey of oral cancer knowledge in the general population in Western Australia, which showed that the available and known information is limited and that greater efforts are needed to educate the population about the disease and the risk factors for its development (23).

In addition, 75.5% of the participants denied the perception of subjective oral disorders, while 24.5% stated that they perceived disorders, most of which could not be associated with possible early symptoms of cancer. After clinical examination, a total of 67 normal findings were confirmed, while a total of 41 oral mucosal changes were noted in 35 subjects. Of the subjects with normal findings, as many as 30 had some subjective symptoms. The fact that we found 14 potentially malignant changes in as many subjects is extremely alarming because 10 denied any symptoms in the oral cavity. Since oral cavity cancer does not produce any characteristic symptoms, especially at the beginning of the disease, regular screening is crucial for timely diagnosis and for preventing severe progression and mortality (4).

Data were also collected on the regularity of examinations of the entire oral cavity. Participants estimated that dental physicians perform a thorough examination in 32.4% of cases, compared with only 6.9% for primary care physicians. According to a study conducted in Iran, the level of training of physicians regarding oral cavity cancer depends on the years of experience and the number of encounters with this disease. Therefore, it is concluded that physicians also need continuous education to refresh their knowledge about oral cavity cancer to increase the possibility of timely detection of potentially pathological lesions (24).

The clinical examinations revealed 14 potentially malignant lesions, ten of which were asymptomatic. Considering that only two of the 14 subjects with suspicious lesions were examined by a dental physician and a general practitioner, it is questionable how many lesions would have been detected in time, thus preventing the severe consequences of the disease. The histopathological findings confirmed the diagnosis of a potentially malignant lesion in ten subjects, whereas it indicated moderate dysplasia in two subjects and carcinoma in situ, i.e., invasive squamous cell carcinoma in one subject (25,26).

This study had several limitations. First, its cross-sectional design precludes the possibility of causal inferences. In addition, the sample size is relatively small, and the study was performed in a single center. As a result, excluding all possible confounding factors that might have affected our results was impossible. Finally, it is possible that some of the participants gave incorrect answers, did not remember, or were overly subjective in their responses to the questionnaire because a self-report questionnaire on a relatively sensitive topic was used.

## Conclusions

Prevalence of oral potentially malignant disorders is considerably higher among wars veterans than in general population. This implies giving special attention to detailed clinical examinations of oral mucosa during their regular dental check-ups.

Dentists and primary care physicians should be educated about the importance of early detection of oral cavity cancer through regular screening and motivated to regularly and in detail examine the entire oral mucosa of all patients regardless of the presence of subjective disorders.

Considering the source of information regarding oral cancer among war veterans was most often the environment, it is imperative to promote the importance of regular screening for early disease detection within the general population.
